# Performance of Thermoplastic Extrusion, Germination, Fermentation, and Hydrolysis Techniques on Phenolic Compounds in Cereals and Pseudocereals

**DOI:** 10.3390/foods11131957

**Published:** 2022-07-01

**Authors:** Luz María Paucar-Menacho, Williams Esteward Castillo-Martínez, Wilson Daniel Simpalo-Lopez, Anggie Verona-Ruiz, Alicia Lavado-Cruz, Cristina Martínez-Villaluenga, Elena Peñas, Juana Frias, Marcio Schmiele

**Affiliations:** 1 Departamento de Agroindustria y Agronomía, Facultad de Ingeniería, Universidad Nacional del Santa, Chimbote 02711, Peru; luzpaucar@uns.edu.pe (L.M.P.-M.); wcastillo@uns.edu.pe (W.E.C.-M.); wsimpalol@uns.edu.pe (W.D.S.-L.); 201612051@uns.edu.pe (A.V.-R.); 201612019@uns.edu.pe (A.L.-C.); 2 Department of Technological Processes and Biotechnology, Institute of Food Science, Technology and Nutrition (ICTAN-CSIC), 28040 Madrid, Spain; c.m.villaluenga@csic.es (C.M.-V.); elenape@ictan.csic.es (E.P.) frias@ictan.csic.es (J.F.); 3 Institute of Science and Technology, Federal University of Jequitinhonha and Mucuri Valleys (UFVJM), MGT-367 Highway-Km 583, No. 5000, Diamantina 39100-000, Brazil

**Keywords:** antioxidant capacity, enzymes, extrusion, grains, microorganism, sprouting

## Abstract

Bioactive compounds, such as phenolic compounds, are phytochemicals found in significant amounts in cereals and pseudocereals and are usually evaluated by spectrophotometric (UV-VIS), HPLC, and LC-MS techniques. However, their bioavailability in grains is quite limited. This restriction on bioavailability and bioaccessibility occurs because they are in conjugated polymeric forms. Additionally, they can be linked through chemical esterification and etherification to macro components. Techniques such as thermoplastic extrusion, germination, fermentation, and hydrolysis have been widely studied to release phenolic compounds in favor of their bioavailability and bioaccessibility, minimizing the loss of these thermosensitive components during processing. The increased availability of phenolic compounds increases the antioxidant capacity and favor their documented health promoting.

## 1. Introduction

Bioactive compounds found in food and with beneficial potential for health include carotenoids, phenolics, alkaloids, nitrogen compounds, steroids, terpenoids, fatty acids, anthraquinones, cardenolides, phlobatannins, and organosulfur compounds, which can promote health singly or in synergy with each other [1,2,3]. Phenolic compounds are natural or synthetic non-nutritive chemical structures represented by hydroxyls and aromatic rings in simple or polymeric forms (can be found in both free and bound states with ester and ether bonds). When present in vegetables, they can be in their free form or complexed to sugars and proteins linked through an OH group (*O*-glycosides) or carbon–carbon bonds (*C*-glycosides) [4]. The free form is commonly found in greater concentrations in fruits and vegetables, while the bound form predominates in grains. Cereals are all monocotyledonous species in the grass family, Poaceae (formally Graminiae), rich in starch. Pseudocereals are dicotyledonous plants belonging to different families; thus, buckwheat (*Fagopyron esculentum*) is placed in the Polygonaceae family, quinoa (*Chenopodium quinoa*) is placed in the Chenopodiaceae, and grain amaranth is placed in the Amaranthaceae family. In cereals and pseudocereals, phenolics are found in insoluble forms through covalent bonds to structural components of the cell wall such as cellulose, hemicellulose, lignin, pectin, carbohydrates, sugars, fatty acids, and proteins. At the same time, free phenolics are present in the vacuoles of plant cells.

The main properties attributed to phenolic compounds in improving the population’s health are protection against non-degenerative chronic diseases and their antioxidant, anti-inflammatory, anticarcinogenic, antimicrobial, anti-apoptosis, and anti-aging capacities. The antioxidant capacity is evaluated through in vitro, ex vivo, and in vivo assays, and increases with more hydroxyl groups, mainly in the ortho position [5]. In addition, some phenolic compounds have antiviral action, such as quercetin, as reported by studies carried out by Ahmed et al. [6], Agrawal et al. [7], and Baroiu et al. [8]. These authors identified pharmacological actions of quercetin against SARS-CoV-2 infections.

However, these compounds are susceptible to degradation and consequently lose their beneficial physiological activities. These losses can be triggered by temperature, exposure to oxygen or modified atmosphere, ionizing and non-ionizing radiation, pressure, and shear force [9]. On the other hand, there are ways to promote the bioavailability of phenolic compounds, highlighting the techniques of germination, fermentation, thermoplastic extrusion, or enzymatic, alkaline, and acid hydrolysis. Occasionally, the extraction can be assisted by microwave, ultrasound, low-frequency ultrasound, infrared, hydrostatic pressure, supercritical fluid, pulsed electric field, ultra-high pressure, and elicitation techniques [10,11,12].

The phenolic compounds of food matrices can be found in free and conjugated forms. When complexed with other food components, bioavailability and are limited [13]. In this sense, the changes promoted by physical, biotechnological, and biochemical processes have gained attention in scientific research and industrial applications. Thus, food matrices with significant phenolic compound levels are remarkable sources of functional ingredients with promising applications in nutraceutical, pharmaceutical, and industrial raw materials, mainly as natural antioxidants. Phytochemical studies have reported antioxidant and antimicrobial activities and anti-inflammatory, anticancer, hypolipidemic, hypoglycemic, immunomodulating, and neuroprotective activities [14,15].

This review addresses the changes promoted by thermoplastic extrusion, germination, fermentation, and acidic, alkaline, or enzymatic hydrolysis on cereals and pseudocereals bioactive compounds in an integrative way. The relevant information that contributes significantly to achieving the goal of this study has been obtained from technical and scientific literature over the last five years.

## 2. Phenolic Compounds Found in Cereals and Pseudocereals

**Figure 1:**
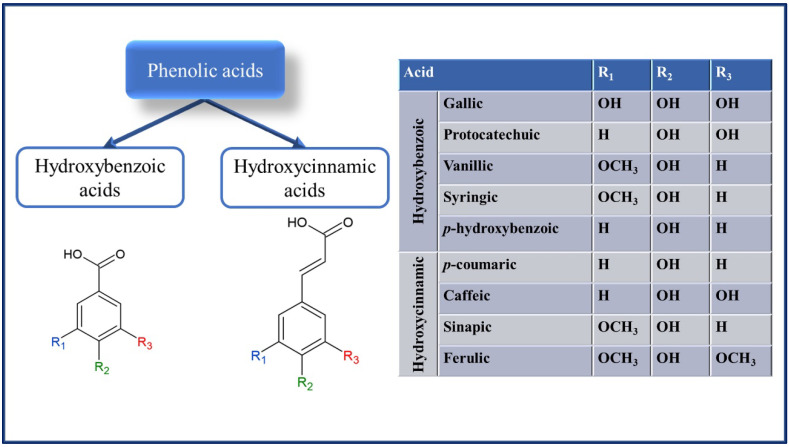
Chemical structure of main phenolic acids found in cereals and pseudocereals.

**Figure 2:**
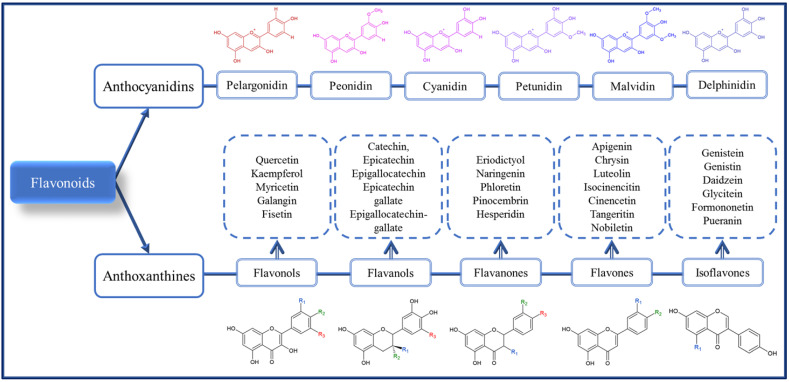
Chemical structure of main flavonoids found in cereals and pseudocereals.

**Figure 3:**
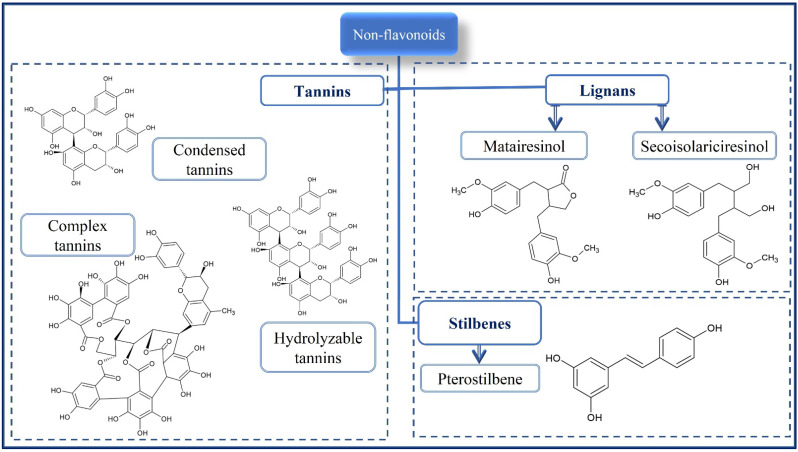
Chemical structure of main non-flavonoids found in cereals and pseudocereals.

Phenolic compounds are phytochemicals found in fruits, vegetables, algae, legumes, pulses, cereals, and pseudocereals. They are secondary metabolites with the primary function of protecting the plant against adverse effects arising from both biotic factors (insects, microorganisms, rodents, and birds) and abiotic factors (humidity, temperature, ultraviolet solar radiation, and nutrients) [9,16]. The phenolic compounds are phenolic acids (Figure 1), flavonoids (Figure 2), and non-flavonoids (stilbenes, chalcones, lignans and tannins) (Figure 3). Tannins are composed of the hydrolysable fraction comprising the esters from ellagic acid (ellagitannins) or gallic acid (gallotannins). The condensed fraction of tannins is comprised of polymeric proanthocyanidins.

Phenolic acids contain a phenolic ring and an organic carboxylic acid function, with a maximum absorption at 280 nm for the C6–C1 skeleton of hydroxybenzoic acid derivatives (gallic, protocatechuic, *p*-hydroxybenzoic, vanillic, and syringic acids) and 320 nm for the C6–C3 skeleton of hydroxycinnamic acid derivatives (*p*-coumaric, ferulic, caffeic, sinapic, chlorogenic and cinnamic acids). In addition, the phenolic ring can stabilize and displace unpaired electrons, imparting an antioxidant property to phenolic acids [17]. The biosynthesis of phenols is derived from several biosynthetic precursors, including some amino acids such as phenylalanine and tyrosine, acetate, pyruvate, malonyl Co, and acetyl CoA through metabolic pathways involving phenylpropanoid, shikimic acid, and pentose phosphate [18].

Phenolic acids include hydroxybenzoic (gallic, protocatechuic, vanillic, syringic) and hydroxycinnamic (coumaric, caffeic, sinapic, ferulic) acids. In addition to hydroxybenzoic and hydroxycinnamic acids, hydroxyphenylacetic and hydroxyphenylpropanoic acids can be found in pseudocereals [19,20]. On the other hand, flavonoids comprise many compounds, especially anthoxanthines (flavonols, flavones, flavanols, flavanones and isoflavonoids), anthocyanidins, and anthocyanins. Some examples of flavonoids are (i) anthocyanidins (cyanidin, petunidin, pelargonidin, delphinidin, peonidin, malvidin) or anthocyanins when glycosylated on C3, forming C3-*O*-glucoside; (ii) flavonols (quercetin, kaempferol, myricetin, galangin, and fisetin); (iii) flavones (apigenin, chrysin, luteolin, isocinencitin, cinencetin, tangeritin, and nobiletin); (iv) flavanols (catechin, epicatechin, epigallocatechin, epicatechin gallate, and epigallocatechin-gallate); (v) flavanones (eriodictyol, naringenin, phloretin, pinocembrin, and hesperidin) [21]; and (vi) isoflavonoids (genistein, genistin, daidzein, tetrahydrodaidzein, glycitein, formononetin, (*S*)-Equol, biochanin A, 8 prenylgenistein, pueranin, genistin-6-*O*-malonate, and genistin-6-*O*-acetate) [22].

## 3. Thermoplastic Extrusion

Thermal processing promotes a series of physical and chemical changes in cereals, such as starch gelatinization, protein denaturation, component interactions, and browning reactions, improving the sensory properties and increasing nutrient availability [23]. It also promotes the inactivation of thermolabile toxic compounds and enzyme inhibitors [24].

Thermoplastic extrusion is classified as a unitary operation that employs a high processing temperature for a short residence time of the material inside the barrel, affecting the food’s microstructure, chemical characteristics, shape, and texture. In addition, thermomechanical actions during extrusion promote the inactivation of enzymes (due to protein denaturation), pathogenic microorganisms, and some antinutritional factors. Thus, polytropic extruders (with heating and cooling control) have been used to produce ready-to-eat products, such as snacks and breakfast cereals, due to the practicality and convenience offered to the consumer. The main challenge for the food industry during the extrusion process is to produce healthy products, maintain their nutritional value, and preserve the bioactive compounds. In addition, the technological and sensory properties of the final product need to be maintained. [25]. However, it is a thermomechanical process in which the operator should be aware of the processing variables, encompassing temperature control, residence time, moisture content, screw speed and configuration, feed rate, and pressure inside the equipment so that the phenolic compounds of the material that will be extruded do not undergo harmful changes.

Thermoplastic extrusion alters antinutritional factors by denaturing proteins during thermomechanical processing and depolymerizing phenolic compounds that interact with proteins, such as condensed tannins [26,27]. In this sense, there is a change in the bioavailability of nutrients and phytochemicals, especially in the fraction of the pericarp of grains, since the phenolic compounds are linked to the fraction of dietary fiber [28].

The effects of extrusion on free and bound phenolic distribution in grains and related products remain controversial. In addition, phenolic bioaccessibility has not been exhaustively studied. Therefore, the effects of extrusion on phenolic profiles and bioaccessibility facilitate the release of phenolics and improve the ability to extract bound phenolics [29].

In sorghum flours, thermoplastic extrusion has been used to alter these antinutritional factors and promote human consumption of sorghum products by denaturing proteins during the process and depolymerizing phenolic compounds that interact with proteins, mainly condensed tannins [30].

The concentrations of phenolic compounds in buckwheat grains vary according to variety, growing season, fertilization conditions, and solar incidence. Common buckwheat showed levels (d.b.) of 5037–11,722 μg/g catechin, 4108–12,858 μg/g chlorogenic acid, 2832–2668 μg/g ellagic acid, 2804–3486 μg/g syringic acid, 743–12,740 μg/g trans-ferulic acid, 279–409 μg/g gallic acid, 66–1354 μg/g *p*-coumaric acid, 26 μg/g cinnamic acid, 21–57 μg/g quercetin, and 12–78 μg/g rutin [31]. Yalcin [32] prepared sheeted noodles containing 60% buckwheat flour and 40% corn starch mixed with hot water (100 °C). The ready-to-eat noodles presented significant levels of phenolic compounds, highlighting levels of 23.39–75.17 μg/g caffeic acid, 11.42–18.99 μg/g 4-hydroxybenzoic acid, 6.91–18.06 μg/g catechin, 3.54–6.11 μg/g rutin, 1.53–2.41 μg/g apigenin, 1.39–4.55 μg/g gallic acid, 1.02–3.45 μg/g 2,5-dihydroxybenzoic acid, 0.57–6.35 μg/g vanillic acid, 0.23–0.80 μg/g ellagic acid, 0.12–0.43 μg/g *p*-coumaric acid, 0.08–3.83 μg/g cinnamic acid, 0.01–0.38 μg/g chlorogenic acid, and 0.01–0.11 μg/g ferulic acid. In addition to the presence of the aromatic ring with sensitivity to high temperatures and mechanical work, functional groups with greater reactivity such as hydroxyl groups make the phenolic compounds more unstable for thermoplastic extrusion processing.

Pigmented rice, such as black rice, has anthocyanins as the main flavonoids, while red rice presents proanthocyanidins as the most significant flavonoids [33]. Meza et al. [28] identified that black rice had 30% more total phenolic compounds and 80% more total flavonoid compounds than red rice. These authors produced breakfast cereal from the flours of both kinds of rice. They found a decrease of 68% and 76% in total phenolic compounds and 70% and 65% in total flavonoid compounds for breakfast cereals from black and red rice, respectively, compared to their unprocessed samples. This reduction affects antioxidant capacities (56–70%), since the phenolics can be degraded due to thermomechanical destruction or changes in molecular structure at high extrusion temperatures. In addition, polymerization can lead to a reduction in chemical reactivity or extractability.

Oliveira et al. [34] highlighted that the rice extrusion with a low residence time of the material inside the extruder barrel was not enough for temperature to impact the phenolic component’s decarboxylation, resulting in the maintenance of these phytochemical compounds. Using a co-rotating twin-screw extruder, Blandino et al. [35] produced snacks from brown, red, and black rice. They found a higher increase in total phenolic compounds and antioxidant capacity in black rice snacks regarding the protocatechuic, hydroxybenzoic, vanillic, caffeic, syringic, *p*-cumaric, and ferulic acids.

However, it should be highlighted that considerable amounts of total phenolic compounds in unprocessed cereal flours can be converted into non-extractable forms. Furthermore, this fraction is bound to other components such as proteins and starchy and non-starch polysaccharides (dietary fibers), with a likely increase in the antioxidant capacity of extrudates. Insoluble phenolic compounds are more resistant to digestion, reaching the colon intact and becoming accessible to the colonic microflora due to possible transformations of polysaccharides or proteins or by the action of some intestinal enzymes [36]. Thus, from a nutritional point of view, these compounds may have direct protective effects on the intestinal mucosa against oxidative stress [28].

Extruded yellow corn grits with conditioning moisture at 13 % and extrusion temperature of 180 °C showed increased levels of *p*-coumaric, vallinic, sinapic, and syringic acids. However, as the extrusion process was severe (low feed moisture and high extrusion temperature), the levels of chlorogenic, rosmarinic, caffeic, ferulic, and salicylic acids showed losses, with chlorogenic acid having only 61.54% of the initial content [37].

Dueñas et al. [38] evaluated the influence of the milling, flaking, and extrusion process on the contents of phenolic compounds in white and brown teff. The authors detected 59 phenolic compounds in the pseudocereals, with 90% composed of *C*-glycosyl flavones, with luteolin and its derivatives responsible for 91–94% of the total flavones. The unit operations changed the phenolic compound profile, increasing *C*-monoglycosylflavones and decreasing acylated derivatives. In the case of brown teff, there was an increase in the content of phenolic compounds when flaking and extrusion technologies were applied. The content of total phenolic compounds increased 78% for thermoplastic extrusion and 269% for flaking. The main compounds that showed increases were the *O*-glycosyl flavones (isoscoparin 2-*O*-rhamnoside-6-*O*-glucoside, luteolin 4-*O*-glucoside-7-*O*-neohesperidoside, luteolin 7-*O*-rutinoside, luteolin 7-*O*-glucoside, and X-methyl-luteolin 7-*O*-rutinoside); *O*-glycosyl flavonols (quercetin 3-*O*-neohesperidoside); *C*-monoglycosyl flavones (luteolin 8-*C*-glucoside, apigenin-8-*C*-glucoside, and 7-methyl-luteolin 8-*C*-glucoside); *O*-glycosyl-*C*-glycosyl flavones (vitexin-*O*-glucoside-*O*-glucoside, 7-*O*-methyl orientin 5-*O*-glucoside, isoorientin 7-*O*-glucoside, isoorientin 7-*O*-neohesperidoside, isovitexin 7-*O*-glucoside, isovitexin 6-*O*-glucoside, 7-*O*-methyl isoorientin, and 6-*O*-glucoside); acetylated *C*-glycosyl flavones (7-*O*-acetyl-orientin); and *C*-glycosylated acylated flavones (isoorientin 6-*O*-(6-*O*-vanilloyl)glucoside and isovitexin 6-*O*-(6-*O*-vanilloyl)glucoside). During heat treatment with low residence time, low moisture content, high pressure, and high shear force, the compartments of the flour fractions break, favoring starch gelatinization and protein denaturation. Thus, the interaction between phenolic compounds with protein bodies and with starchy and non-starch fractions can be thermodynamically disadvantaged, breaking chemical bonds and facilitating the extraction of these bioactive compounds. Table 1 presents an overview of the effect of the extrusion process on phenolic compounds. Thermoplastic extrusion should be realized conveniently to favor the increase of free bioactive compounds but so that the quality of the other components is not affected, and the significant loss of thermosensitive components occurs. The thermoplastic extrusion process acts under high temperatures, pressures, and shear forces simultaneously, and thus the phenolic compounds have subtle increments concerning increase during processing. Different authors have reported contrary effects on phenolic compound content on extruded products. These effects come from the complexity of the extrusion process, since several parameters may have an influence on phytochemical compounds such as zone temperatures, feed rate, feed moisture, pressure, barrel and screw configuration, screw speed, residence time, shape, and opening die.

## 4. Germination

The germination of grains can be considered to be a simple, inexpensive, and effective way to improve the nutritional value of cereals and pseudocereal kernels. Germination is widely used to enhance cereal quality, soften the kernel structure, and increase bioactive compounds in the cereal seeds. For germination, the grains must be sanitized and soaked in water in the presence of oxygen and submitted to sprouting under controlled temperature, relative humidity, and light/dark conditions wherein endogenous enzymes are synthesized/released. The main challenge in the cereal and pseudocereals germination process is inadequate sanitization, as it will result in the development of microorganisms, resulting in exogenous enzymes in the sprouting process. In addition, the grain soaking process should be performed adequately, as the seed embryo must maintain its metabolic activities so that the synthesis and release of endogenous enzymes occurs efficiently. It has been reported that germination improves the nutritional value of the biochemical and biofunctional compounds released through the hydrolysis process promoted by the activated endogenous enzymes related to the degradation of reserve materials used for respiration and synthesis of new cell constituents for developing embryo in the seed [45,46].

The germination process has been studied in several types of grains, and its beneficial effects have been reported, such as increased bioactive compounds and improved protein digestibility [47]. For example, significant increases in *p*-hydroxybenzoic acid, *p*-hydroxybenzaldehyde, vanillin, *p*-coumaric acid, ferulic acid, and epigallocatechin were found by Chu et al. [48] during the germination of wild rice for up to 120 h.

Paucar-Menacho et al. [49] applied the response surface methodology to study the effect of time (12–72 h) and temperature (12–28 °C) of amaranth germination, evaluating the performance of phenolic compounds. The best result was obtained after 63 h of germination at 26 °C. The authors reported a 73.81-fold increase in total phenolic compound content, a 5.39-fold increase in total flavonoid content, and an 85.83-fold rise in non-flavonoid compound content. Sprouting in the optimal germination conditions mainly enhanced the concentrations of 4-*O*-caffeoylquinic, 4-*O*-feruloylquinic acids and quercetin-3-*O*-rutinoside. The phenolic acids (hydroxybenzoic and hydroxycinnamic) were the most abundant in the germinated grains, corresponding to between 85 and 99% for the experimental design tests. Amaranth presents high concentrations of hydroxybenzoic acid, both in the free form (vanillic acid) and in the esterified form with sugars (pentoses and hexoses), representing approximately half of the phenolic compounds. Cinnamic acids represent around 34% of phenolic compounds and are mostly linked to hydroxy and quinic acids.

Sharma et al. [50] studied the effect of soaking time, soaking temperature, and time of germination on Kodo millet seeds and observed that the ungerminated grain had a total phenolic content of 54.54 mg of gallic acid equivalent per 100 g of sample. The best germination condition resulted in 83.01 mg of gallic acid equivalent per 100 g of sample. In addition, the total antioxidant capacity showed an increase of 52% and the hydrogen peroxide scavenging activity (expressed in millimole equivalents of Trolox/g) increased by 70%.

Kaempferol, rutin, quercetin, orientin, vitexin, quercetin, iso-orientin, and isovitexin are flavonoids that have been found to be increased in level through the germination process in buckwheat. Dumitru et al. [51] submitted Achenes buckwheat to the germination process (23–25 °C for seven days) and obtained an increase in total phenolic compounds of 4.6 fold (4.91 and 22.57 mg GAE/g d.b., for ungerminated and germinated buckwheat seeds, respectively). The total flavonoid content increase was slightly higher (5.4 fold), such as the results obtained for 2,2-diphenyl-1-picrylhydrazyl (DPPH) radical scavenging activity assay 5.8 times higher for the germinated buckwheat. The authors identified 18 phenolic compounds in the study, with the highest levels quantified being vitexin (5115.31 µg/g), orientin (2361.50 µg/g), isoorientin (1475.85 µg/g), coumestrol (917.71 µg/g), and quercetin arabinoside (885.17 µg/g), increasing to 3185, 1220, 1191, 2562 and 286%, respectively, compared to the ungerminated grain. The lowest increase was found in catechin levels (56%), such as reducing hydroxysecoisolariciresinol (lignan) and epigallocatechin levels. Other phenolic compound identified in germinated buckwheat were rutin, epigallocatechingallate, 4 gallocatechingallate, secoisolariciresinol, isorhamnetin glucuronide, quercetin ramnosid (quercitrin), quercetin (quercetin-3-*O*-rhamnoside), isorhamnetin, luteolin, and apigenin.

The germination time (12–72 h) and temperature (12–28 °C) of purple maize were evaluated by Paucar-Menacho et al. [52] through a central composite rotational design. Germination at 26 °C for 63 h showed the best increase in phenolic compounds. The seeds germinated in the optimal condition presented anthocyanins as the predominant class, especially cyanidin, pelargonidin, peonidin glycosides, and their malonyl derivatives. Hydroxycinnamic acids derivatives and flavonols were detected as minor compounds.

The elicitation technique has been combined with germination biotechnology to favor the release of bound phenolic compounds. Gómez-Velázquez et al. [53] applied salicylic acid and hydrogen peroxide to improve the release of phenolic compounds and antioxidant properties of germinated chia grain. The germination process was carried out at 26.5 °C for 178 h and produced a 64% germination and a sprout length of 56 mm. The elicitation with H_2_O_2_ (20 mM) enhanced the germination (75%) and the sprout length (59 mm). In contrast, the use of salicylic acid at concentrations of 1 and 2 mM increased the content of total phenolic compounds up to 65.5–73.5%, leading to an increase in the antioxidant capacities of all chia sprouts evaluated by DPPH (86%) and 2,2′-azinobis-(3-ethylbenzothiazoline-6-sulfonic acid) (ABTS) (61%) assay when 1 M of salicylic acid was applied. Elicitors could activate enzymes and defense genes to increase the accumulation of phenolic compounds, improving antioxidant capacities and obesity-related oxidative stress in serum and urine compared to non-elicited sprouts.

## 5. Fermentation and Enzymatic Hydrolysis

**Figure 4:**
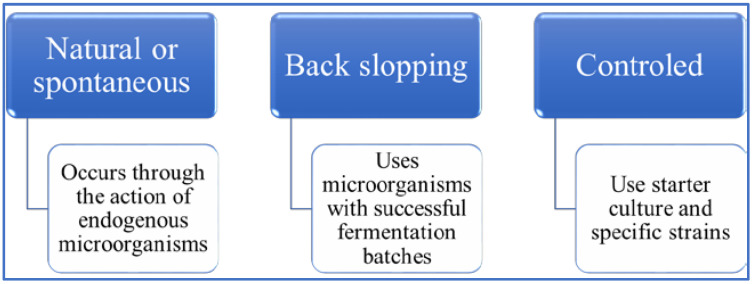
Conventional techniques for fermentation processes.

Fermentation comprises conventional techniques (Figure 4) involving the conversion or modification of substrates promoted by the metabolic activity of specific microorganisms. This microbial action promotes positive appearance, color, flavor, texture, technological functionalities, nutritional composition, nutrient bioavailability, and physiological effects. In addition to compounds beneficial to the diet, fermentation may also be responsible for reducing compounds that are harmful to human health [54].

Enzymatic hydrolysis, although more expensive than the fermentation technique, is more advantageous due to the specificity of the biochemical process. Furthermore, in the case of fermentation by microorganisms, other enzymes can be released during the fermentation process, as well as secondary compounds that may remain after the fermentative process.

Most of the time, flours, semolina, and grits are used for the fermentation of cereals. The whole grain fermentation process is slow due to the protection of the grains’ pericarp, tegument, and aleurone layer, reducing the diffusion of nutrients such as amino acids and sugars necessary for the growth of microorganisms. In addition to milling, raw material extrusion before fermentation can be an interesting strategy to improve the fermentation process. Among the leading food products based on cereals produced by fermentation by microorganisms (mainly lactic acid bacteria) with positive results for the bioavailability of phenolic compounds are sourdoughs, tempe, koji, ting, and dawadawa (an African fermented condiment). However, several other fermented products can be found in the literature and in the consumer market [21].

The fermentation process in cereals and pseudocereals affects phenolic compounds, turning the condensed fraction (usually esterified or etherified) into the free form through bioconversion, favoring extraction, bioavailability, and bioaccessibility. It is important to highlight that the during food processing and preparation (cooking, baking, and frying, among others) these free forms could be destroyed and no longer be bioavailable. In addition, there are modifications or the formation of other conjugates, glycosides, or related monomers or polymers [55]. The fermentation process for cereals and pseudocereals generally occurs in a solid state, but other forms such as semi-solid or submerged state can also be applied [56].

The transformation of the condensed fraction into a free one is favored by the metabolic activities of microorganisms, inducing the structural breakdown of the cell wall and promoting the synthesis of several bioactive compounds in more active forms. An increase in the antioxidant capacity occurs with the release of phenolic compounds through enzymatic hydrolysis during fermentation. In addition to phenolic compounds, other components with antioxidant capacity can be released, such as peptides and polypeptides, vitamins, and exopolysaccharides from the enzymatic action synthesized by bacteria and fungi, resulting in a protective effect against oxidation. Furthermore, phenolic compounds modify cell signaling processes, donate or transfer electron/hydrogen atoms to free radicals, activate endogenous antioxidant mechanisms, increase antioxidant capacity levels, and act as chelators for trace metals involved in free radical protection [57].

Hydrolysis occurs through the action of enzymes synthesized and released during fermentation. Among these enzymes are proteases, amylases, *β*-glucosidase, amyloglucosidase, xylanases, hemicellulases, cellulases, tannases, and phytases. Furthermore, other enzymatic reactions involve the activities of carboxylases, decarboxylases, esterases, oxidases, reductases, and hydrolases. Some identified conversions were the following transformations: (i) from caffeic acid into dihydrocaffeic acid, 4 ethylcatechol, and vinylcatechol; (ii) from ferulic acid to dihydroferulic acid; and (iii) naringenin-7-*O*-glucoside to naringenin. This metabolism can be influenced by the intrinsic factors of food matrices, such as the metabolized substrate. Thus, the metabolic pathway can change, and enzymatic activities can alter from decarboxylase to reductase, reaching hydrolytic activities [56,58].

Although lactic acid bacteria are mainly responsible for the increase in the content of phenolic compounds, co-fermentation with yeasts improves the conversion of the resulting free ferulic acid into dihydroferulic acid and volatile metabolites such as vinyl-guaiacol and ethyl-guaiacol. This behavior suggests that the metabolism of phenolic acids in yeast is enhanced by co-fermentation due to synergism between metabolic activities [59].

Khan et al. [60] studied the effect of co-fermentation between *L. plantarum*, *L. fermentum* and *S. cerevisiae* for 24 h at 37 °C using extruded and saccharified brown rice (amylolytic enzymes) as substrate in submerged fermentation. The results showed that the combination of extrusion, saccharification, and co-fermentation operations increased in free (90.84%), conjugated (105.46%), bound (85.41%), and total (93.32%) phenolic compounds. Concerning flavonoids, they verified a similar behavior, with an increase in free (74.26%), conjugated (49.07%), bound (65.29%), and total (61.63%) flavonoid compounds. The main phenolic acids identified were ferulic, gallic, syringic, *p*-coumaric, vanillic, chlorogenic, cinnamic, and syringic, and the flavonoids were epicatechin, quercetin, and kaempferol. In addition, the total antioxidant capacity was evaluated by the ORAC (oxygen radical absorbance capacity) method and showed values of 33.49 µmol of Trolox equivalent/g for the sample without co-fermentation and 62.90 µmol of Trolox equivalent/g when applying the joint action of the three microorganisms, which increased by 87.82%.

Zielinski et al. [61] evaluated the effect of 14 strains of *Lactobacillus* in buckwheat flour fermentation using the flour fermented in water biscuits on the total phenolic compounds. The authors state that all strains of lactic acid bacteria had a beneficial effect on the release of total phenolic compounds from fermented flours (to a greater or lesser degree). The application of *L. plantarum* IB resulted in higher levels of total phenolic compounds in flour and cookies due to better decarboxylation and reduced enzymatic activity. The most significant increases in phenolic acid through the flour fermentation process were obtained for sinapic and caffeic acids. In contrast, for the flavonoids, the fermentation favored an increase in the content of luteolin, apigenin and epicatechin, with the total flavonoid compounds increasing by 7.59%.

The biotechnological process of natural fermentation was investigated in millet sourdough [62] and refined or whole wheat flour [63,64]. The fermentation of pearl millet flour was induced by a starter culture containing yeast, *L. sanfranciscensis*, and *L. pentosus*. In the six samples of sourdoughs produced, eight phenolic acids derived from cinnamic acid were identified (N2,N4-dicaffeoylspermidine, *p*-coumaric acid, ferulic acid, ferulic acid rhamnoside, ferulic acid rhamnoside isomer, isoferulic acid, methylhydroxycinnamate, and methyl ferulate) and four flavonoid compounds (luteolin-(7-*O*-glucopyranosyl)-8-*C*-glucopyranoside, vicenin II, vitexin 2″-*O*-rhamnoside, and vitexin). The sourdough presented a free phenolic compound content between 48.2 and 127.4 mg/100 g and a total content between 66.5 and 149.3 mg/100 g. The increase in antioxidant capacity was also verified with the spontaneous fermentation of refined and whole wheat flour. The increase was more significant for refined wheat flour (51.02%) than for whole wheat flour (32.09%). The metabolism of lactic bacteria increases the antioxidant capacity of bread made with sourdough due to the synthesis or release and greater extractability of phenolic compounds that can be attributed to the higher acidity in the medium and enzymatic hydrolysis. The rate of reduction in the pH of the medium reflects inversely on the release of phenolic compounds, with slow acidification being advantageous as it prolongs the action of microorganisms (lactic acid bacteria and yeasts).

Chiş et al. [65] evaluated the content of total flavonoid compounds in quinoa flour fermented either spontaneously or using *L. plantarum* ATCC 8014 as a starter culture. Fermentation took place at 37 °C for 24 h. Biochemical reactions promoted by the *Lactobacillus* strain are responsible for favoring the enzymatic action and hydrolysis of glycosides flavonoids and isoflavone aglycones. When using the starter culture, the content of total flavonoid compounds was 1551 mg quercetin per gram of flour, while for spontaneous fermentation, the value reached was 757 mg quercetin per gram of flour. The main phenolic compounds found in quinoa are ecdysteroids, phenolic acids, and flavonoids such as kaempferol and quercetin.

A mix-culture composed of *M. anka* GIM 3.592, *S. cerevisiae* GIM 2.139 and *B. subtilis* 784 was used by Luo et al. [66] in the fermentation of corn seeds. The metabolic action of microorganisms resulted in a significant increase in chlorogenic, *p*-hydroxybenzoic, caffeic, vanillic, *p*-coumaric, sinapic, and ferulic acids as well as rutin and quercetin. The highest content was found for chlorogenic acid (2920.66 mg/kg).

*M. anka* GIM 3.592 and *Bacillus subtilis* 784 were also applied by Chen et al. [67] in oat grains using solid-state fermentation at 37 °C for 12 days and stirred at 180 rpm. For free phenolic acids, the highest increase was found for chlorogenic acid (47.31 fold) and ferulic acid (28.22 fold). Regarding bound phenolics, *p*-coumaric acid, sinapic acid and ferulic acid showed the most significant increases, with 44.19, 51.38, and 40.35 times, respectively. Conjugated phenolic content ranged from 20.6 to 438.42 mg/kg, with chlorogenic acid content increasing nearly 100 fold. The quantification of total phenolic compounds showed an increase of 26.36 times for free (30.32 to 829.49 mg/kg), 20.28 times for conjugates (20.60 to 438.42 mg/kg) and 52.07 fold for bound (9.87 to 523.81 mg/kg). The best antioxidant capacity increased by 93.75% for the DPPH assay between the control sample and the bound fraction and 147.44% for the ABTS assay between the control sample and the conjugated fraction. This behavior corroborates the difference between antioxidant activity and antioxidant capacity, as not all fractions that present conditions for scavenging free radicals can do so satisfactorily.

Enzymatic hydrolysis performed by biocatalysts such as cellulases, amylase, hemicellulases, and pectinase plays an essential role in releasing bound phenolic acids from the cell wall of cereals. The synergistic effect between cellulase action from *A. niger* and solid-state fermentation with *M. anka* GIM 3.592 in oat grains was studied by Bei et al. [68]. Samples were incubated at 30 °C for 14 days. The most significant increases in phenolic compounds were obtained after six days of biotechnological action, and the increases in soluble and insoluble phenolic contents were 76.76 and 23.23%, respectively. The content of total phenolic compounds in the control sample was 0.44 mg/g and 5.51 mg/g after six days of fermentation, increasing 11.52 fold. This result implies that the combined effect between the enzyme and the microorganism favored the release and extraction of soluble phenolics and the synthesis of new phenolic compounds by *Monascus* fungi via the polyketide pathway.

Physical and biotechnological processes have replaced acid and alkaline hydrolysis to release and extract bound/conjugated phenolic compounds in cereals and pseudocereals. In recent years, the concern with reducing chemical reagents has created an increased interest in research of technologies that are cheaper without polluting the environment and do not require further treatment of the generated effluent, but are simultaneously technologically viable.

## 6. Conclusions and New Trends

The search for high-quality functional foods has been widely explored by the scientific community and the food industry to meet the needs of consumers. Phenolic compounds play an essential role in foods in terms of health-beneficial properties. Therefore, chemical processes are increasingly being replaced by physical processes such as thermoplastic extrusion and biotechnological processes such as germination, fermentation, and enzymatic hydrolysis. The highest increase in phenolic compound content was observed when the germination process was applied, followed by enzymatic hydrolysis, fermentation, and finally, the thermoplastic extrusion technique. The better performance of the germination process is due to the use of low temperatures and controlled relative humidity and light conditions.

Furthermore, during the germination process, there is a synergism of the enzymatic hydrolysis effect, resulting in a better performance of the biotechnological process of germination. Combining these processes presents itself as promising for synergistic action in the release and extraction of phenolic compounds from food matrices such as cereals and pseudocereals. The economic aspects of the processes covered may vary depending on the technique applied, the throughput capacity, the availability of raw material, and the added value of the final product. However, it is emphasized that thermoplastic extrusion is a process that does not generate effluents and is considered environmentally friendly. On the other hand, the germination process is an increasingly used tool because it is a low-cost biotechnological process. Fermentation and enzyme processes are more costly among the topics covered. However, all techniques do not generate chemical waste, in addition to valuing local development, agribusiness, sustainability, preservation of the environment, and the availability of raw materials or products with a more significant health claim.

However, it is vital to maintain food’s technological and sensory properties so that the consumer does not notice changes in palatability, well-being, and wellness. Therefore, providing safe and healthy food and correctly processing, using innovative, economical, and eco-friendly tools have been one of the main goals of the academic community. Therefore, new strategies must be developed ethically and transparently with research, industry, and government agencies. Finally, genetic engineering promises to improve the biodiversity available in the environment to increase the microorganism or enzymatic performance. Herewith, de novo biosynthesis is innovative to achieve the goal of producing complex molecules from simple structures.

## Figures and Tables

**Table 1:** Total phenolic compounds (TPC), free phenolic compounds (FPC), and bound phenolic compounds (BPC) from raw material and extrudates from some cereals and pseudocereals under different feed moisture and extruder temperatures.

Raw Material	Extrusion Conditions	TPC mg GAE/100 g	FPC mg GAE/100 g	BFC mg GAE/100 g	Reference
Sorghum bran	Unextruded	2941.65	638.14	2303.52	[39]
	Mildest extrusion condition (feed moisture—30% and the 4th extrusion zone temperature—140 °C)	3429.23	742.27	2686.96	
	Most severe extrusion condition (feed moisture—26.46% and the 4th extrusion zone temperature—174.14 °C)	2712.30	644.93	2470.64	
Yellow corn grits	Unextruded	180.37	-	-	[37]
	Extruded with feed moisture (13%) and extrusion die temperature (180 °C)	21.63	-	-	
Whole corn flour	Unextruded	40.00	17.50	23.50	[40]
	Extruded with feed moisture (14%) and extrusion die temperature (120 °C)	101.70	23.50	78.20	
Barley flour (BH-885)	Unextruded	392.20	-	-	[41]
	Extruded with feed moisture (30%) and extrusion die temperature (150 °C)	327.70	-	-	
	Extruded with feed moisture (13%) and extrusion die temperature (180 °C)	275.60	-	-	
Defatted rice bran	Unextruded	206.80	19.06	187.70	[29]
	Extruded with feed moisture (25%) and extrusion zones temperature (70, 98 and 134 °C)	320.02	47.39	272.64	
Sorghum flour with tannins (cultivar 9929026) *	Unextruded	317.00	7.83	82.04	[42]
	Extruded with feed moisture (16%) and extrusion zones temperature (60, 120 and 140 °C)	189.00	8.35	57.00	
Sorghum flour without tannins (cultivar 2012038) *	Unextruded	139.00	6.13	85.59	
	Extruded with feed moisture (16%) and extrusion zones temperature (60, 120 and 140 °C)	124.00	8.02	91.37	
Red quinoa flour	Unextruded	1806.70	221.50	1585.20	[43]
	Extruded with feed moisture (25%) and extrusion die temperature (120 °C)	1299.40	458.60	840.80	
	Extruded with feed moisture (25%) and extrusion die temperature (140 °C)	1182.90	410.40	772.50	
	Extruded with feed moisture (25%) and extrusion die temperature (160 °C)	1253.50	472.50	781.00	
	Extruded with feed moisture (25%) and extrusion die temperature (180 °C)	1233.40	431.70	801.70	
Broomcorn millet flour	Unextruded	10.77	-	-	[44]
	Extruded with feed moisture (20%) and extrusion die temperature (110 °C)	19.24	-	-	

* Free and bound phenolic acids is the sum of *p*-coumaric, *o*-coumaric, ferulic, sinapic, gallic, vanillic, chlorogenic, caffeic, and syringic acids.

## Data Availability

Not applicable.
